# Crystal structure of 2,6-dimethyl-4-pyridone hemihydrate

**DOI:** 10.1107/S2056989015012402

**Published:** 2015-07-04

**Authors:** Dalena M. Nguyen, Vasumathi Desikan, James A. Golen, David R. Manke

**Affiliations:** a Department of Science & Math, Massasoit Community College, 1 Massasoit Boulevard, Brockton, MA 02302, USA; b Department of Chemistry and Biochemistry, University of Massachusetts Dartmouth, 285 Old Westport Road, North Dartmouth, MA 02747, USA

**Keywords:** crystal structure, hydrogen bonding, pyridones

## Abstract

The title compound (systematic name: 2,6-dimethyl-1*H*-pyridin-4-one hemihydrate), C_7_H_9_NO·0.5H_2_O, has a single planar mol­ecule in the asymmetric unit with the non-H atoms possessing a mean deviation from planarity of 0.021 Å. There is also half of a water mol­ecule present in the asymmetric unit. In the crystal, infinite (001) sheets are formed by N—H⋯O and O—H⋯O hydrogen bonds.

## Related literature   

For the crystal structure of the parent 4-pyridone, see: Jones (2001 ▸); Tyl *et al.* (2008 ▸). For the title compound bound to zirconium, see: Castillo *et al.* (1987 ▸). For the structure of a chloro-substituted variant of the title compound, see: Boer (1972 ▸).
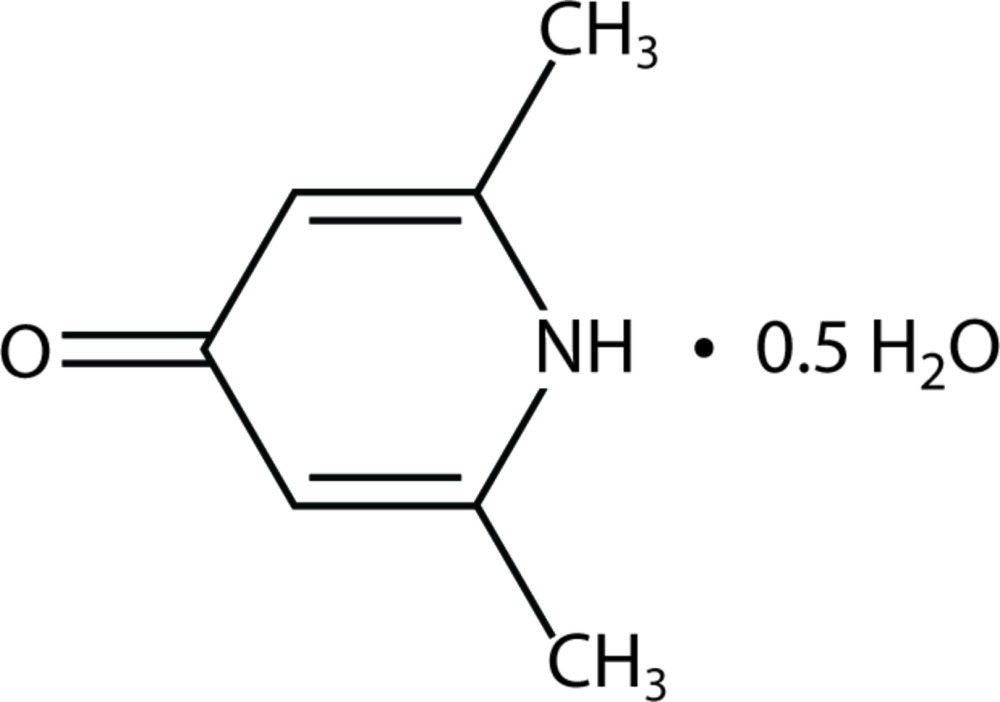


## Experimental   

### Crystal data  

C_7_H_9_NO·0.5H_2_O
*M* *_r_* = 132.16
Orthorhombic, 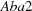

*a* = 12.4859 (17) Å
*b* = 14.3697 (19) Å
*c* = 7.732 (1) Å
*V* = 1387.3 (3) Å^3^
*Z* = 8
Cu *K*α radiation
μ = 0.73 mm^−1^
*T* = 120 K
0.5 × 0.1 × 0.1 mm

### Data collection  

Bruker Venture D8 CMOS diffractometer
Absorption correction: multi-scan (*SADABS*; Bruker, 2014 ▸) *T*_min_ = 0.554, *T*_max_ = 0.754
11786 measured reflections
1366 independent reflections
1352 reflections with *I* > 2σ(*I*)
*R*_int_ = 0.060

### Refinement  

*R*[*F* ^2^ > 2σ(*F* ^2^)] = 0.030
*wR*(*F* ^2^) = 0.078
*S* = 1.09
1366 reflections
95 parameters
3 restraints
H atoms treated by a mixture of independent and constrained refinement
Δρ_max_ = 0.15 e Å^−3^
Δρ_min_ = −0.19 e Å^−3^
Absolute structure: Flack *x* determined using 611 quotients [(*I* ^+^)-(*I* ^-^)]/[(*I* ^+^)+(*I* ^-^)] (Parsons *et al.*, 2013 ▸.
Absolute structure parameter: 0.05 (12)

### 

Data collection: *APEX2* (Bruker, 2014 ▸); cell refinement: *SAINT* (Bruker, 2014 ▸); data reduction: *SAINT*; program(s) used to solve structure: *SHELXS97* (Sheldrick, 2008 ▸); program(s) used to refine structure: *SHELXL2014* (Sheldrick, 2015 ▸); molecular graphics: *OLEX2* (Dolomanov *et al.*, 2009 ▸); software used to prepare material for publication: *OLEX2* and *publCIF* (Westrip, 2010 ▸).

## Supplementary Material

Crystal structure: contains datablock(s) I. DOI: 10.1107/S2056989015012402/ff2139sup1.cif

Structure factors: contains datablock(s) I. DOI: 10.1107/S2056989015012402/ff2139Isup2.hkl

Supporting information file. DOI: 10.1107/S2056989015012402/ff2139Isup3.cml

**Fig. 1.**
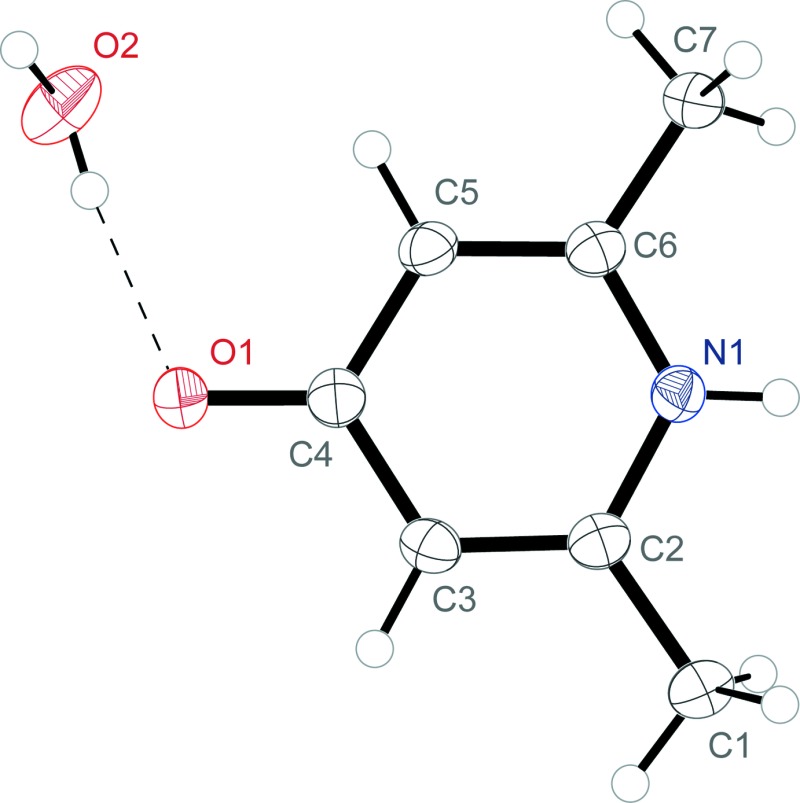
Molecular structure of the title compound, showing the atom-labeling scheme. Displacement ellipsoids are drawn at the 50% probability level. H atoms are drawn as spheres of arbitrary radius.

. DOI: 10.1107/S2056989015012402/ff2139fig1.tif
Mol­ecular structure of the title compound, showing the atom-labeling scheme. Displacement ellipsoids are drawn at the 50% probability level. H atoms are drawn as spheres of arbitrary radius.

**Fig. 2.**
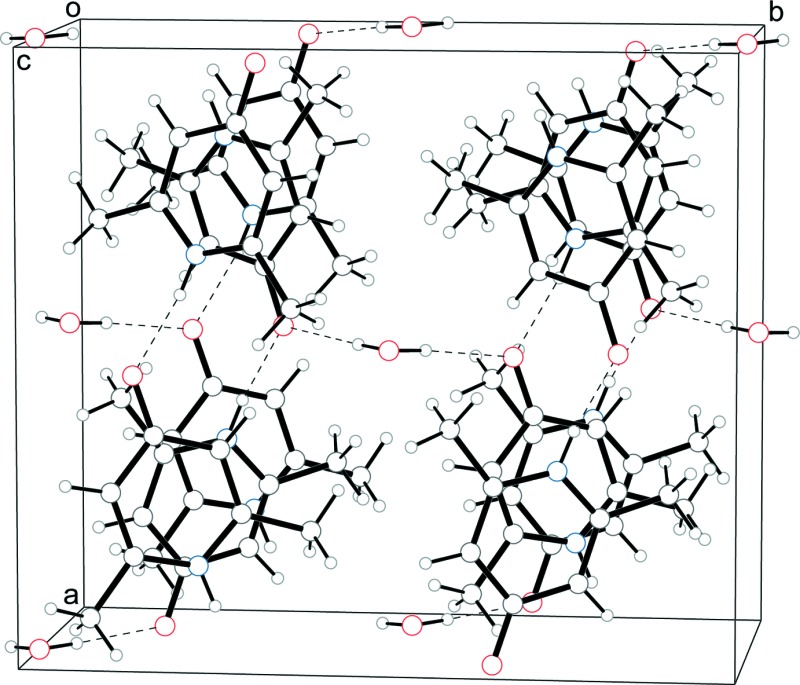
Molecular packing of the title compound with hydrogen bonding shown as dashed lines.

. DOI: 10.1107/S2056989015012402/ff2139fig2.tif
Mol­ecular packing of the title compound with hydrogen bonding shown as dashed lines.

CCDC reference: 1409189

Additional supporting information:  crystallographic information; 3D view; checkCIF report

## Figures and Tables

**Table 1:** Hydrogen-bond geometry (, )

*D*H*A*	*D*H	H*A*	*D* *A*	*D*H*A*
O2H2O1	0.86(1)	1.96(1)	2.8174(17)	173(2)
N1H1O1^i^	0.87(1)	1.86(1)	2.7154(18)	166(3)

Symmetry code: (i) 
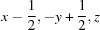
.

## supplementary crystallographic information

### S1. Comment

The structure of the title compound shows that 2,6-di­methyl-4-hy­droxy­pyridine takes the pyridone form in the solid state. Though the title compound has not been crystallographically characterized, a structure of the molecule bound to zirconium has been reported (Castillo *et al.*, 1987) with similar bond distances and angles observed. The parent 4-pyridone has been structurally characterized (Tyl *et al.*, 2008) and exhibits similar chains linked by head-to-tail N–H···O hydrogen bonds, which are also observed in the close derivative Clopidol (Boer, 1972). While the title compound crystallizes in a 2:1 ratio of pyridone to water, the hydrate of the parent molecule crystallizes in a 5:6 ratio (Jones, 2001).

The molecular structure of the title compound is shown in Figure 1. The molecule is near planar with the non-hydrogen atoms possessing a mean deviation from the plane of 0.021 Å. Head-to-tail N1–H1···O1 hydrogen bonding leads to chains that are further linked by O2–H2···O1 hydrogen bonds with water molecules in the crystal to form two-dimensional (001) sheets. The packing of the title compound indicating hydrogen bonding is shown in Figure 2.

### S2. Experimental

A commercial sample (Oakwood Chemical) of 4-hy­droxy­pyridine was used for the crystallization. Crystals suitable for single crystal X-ray analysis were grown by slow evaporation of a methanol solution.

### S3. Refinement

All non-hydrogen atoms were refined anisotropically (*XL*) by full matrix least squares on F^2^. Hydrogen atoms H1 and H2 were found from a Fourier difference map. H1 was refined at a fixed distance of 0.87 (0.005) Å and an isotropic displacement parameter 1.20 times *U*_eq_ of the parent N atom. H2 was refined at a fixed distance of 0.86 (0.005) Å and an isotropic displacement parameter 1.50 times *U*_eq_ of the parent O atom. The remaining hydrogen atoms were placed in calculated positions and then refined with riding models with C—H lengths of 0.98 Å for (CH_3_) and 0.95 Å for (CH) with isotropic displacement parameters set to 1.20 times *U*_eq_ of the parent C atoms.

### Crystal data

**Table:**

C_7_H_9_NO·0.5H_2_O	*F*(000) = 568
*M**_r_* = 132.16	*D*_x_ = 1.266 Mg m^−^^3^
Orthorhombic, *A**b**a*2	Cu *K*α radiation, λ = 1.54178 Å
Hall symbol: A 2 -2ac	Cell parameters from 9829 reflections
*a* = 12.4859 (17) Å	θ = 7.1–72.4°
*b* = 14.3697 (19) Å	µ = 0.73 mm^−^^1^
*c* = 7.732 (1) Å	*T* = 120 K
*V* = 1387.3 (3) Å^3^	NEEDLE, colourless
*Z* = 8	0.5 × 0.1 × 0.1 mm

### Data collection

**Table:**

Bruker Venture D8 CMOS diffractometer	1366 independent reflections
Radiation source: microfocus Cu	1352 reflections with *I* > 2σ(*I*)
HELIOS MX monochromator	*R*_int_ = 0.060
φ and ω scans	θ_max_ = 72.4°, θ_min_ = 7.1°
Absorption correction: multi-scan (*SADABS*; Bruker, 2014)	*h* = −15→15
*T*_min_ = 0.554, *T*_max_ = 0.754	*k* = −17→17
11786 measured reflections	*l* = −9→9

### Refinement

**Table:**

Refinement on *F*^2^	Hydrogen site location: mixed
Least-squares matrix: full	H atoms treated by a mixture of independent and constrained refinement
*R*[*F*^2^ > 2σ(*F*^2^)] = 0.030	*w* = 1/[σ^2^(*F*_o_^2^) + (0.0453*P*)^2^ + 0.3383*P*] where *P* = (*F*_o_^2^ + 2*F*_c_^2^)/3
*wR*(*F*^2^) = 0.078	(Δ/σ)_max_ < 0.001
*S* = 1.09	Δρ_max_ = 0.15 e Å^−^^3^
1366 reflections	Δρ_min_ = −0.19 e Å^−^^3^
95 parameters	Absolute structure: Flack *x* determined using 611 quotients [(*I*^+^)-(*I*^-^)]/[(*I*^+^)+(*I*^-^)] (Parsons *et al.*, 2013.
3 restraints	Absolute structure parameter: 0.05 (12)

### Special details

**Table:**

Experimental. Absorption correction: SADABS-2014/4 (Bruker,2014) was used for absorption correction. wR2(int) was 0.1440 before and 0.0881 after correction. The Ratio of minimum to maximum transmission is 0.7350. The λ/2 correction factor is 0.00150.
Geometry. All e.s.d.'s (except the e.s.d. in the dihedral angle between two l.s. planes) are estimated using the full covariance matrix. The cell e.s.d.'s are taken into account individually in the estimation of e.s.d.'s in distances, angles and torsion angles; correlations between e.s.d.'s in cell parameters are only used when they are defined by crystal symmetry. An approximate (isotropic) treatment of cell e.s.d.'s is used for estimating e.s.d.'s involving l.s. planes.

### Fractional atomic coordinates and isotropic or equivalent isotropic displacement parameters (Å^2^)

**Table:**

	*x*	*y*	*z*	*U*_iso_*/*U*_eq_	
O1	0.47723 (9)	0.33620 (8)	0.49203 (19)	0.0287 (3)	
O2	0.5000	0.5000	0.6889 (3)	0.0399 (5)	
N1	0.16883 (11)	0.24949 (11)	0.4446 (2)	0.0215 (3)	
C3	0.35308 (14)	0.22232 (12)	0.3962 (2)	0.0234 (4)	
H3	0.4083	0.1841	0.3504	0.028*	
C2	0.24877 (14)	0.19343 (12)	0.3853 (2)	0.0222 (4)	
C4	0.38007 (12)	0.30864 (12)	0.4748 (2)	0.0226 (4)	
C7	0.09367 (14)	0.39066 (13)	0.5690 (3)	0.0265 (4)	
H7A	0.0630	0.4210	0.4669	0.040*	
H7B	0.1158	0.4381	0.6527	0.040*	
H7C	0.0399	0.3499	0.6218	0.040*	
C6	0.18900 (13)	0.33399 (11)	0.5164 (2)	0.0217 (4)	
C1	0.21576 (15)	0.10115 (12)	0.3125 (3)	0.0270 (4)	
H1A	0.1736	0.0672	0.3988	0.040*	
H1B	0.2797	0.0650	0.2828	0.040*	
H1C	0.1724	0.1109	0.2085	0.040*	
C5	0.29237 (13)	0.36395 (12)	0.5339 (2)	0.0229 (4)	
H5	0.3061	0.4226	0.5862	0.027*	
H2	0.4956 (19)	0.4524 (12)	0.621 (3)	0.034*	
H1	0.1043 (9)	0.2265 (14)	0.444 (4)	0.027*	

### Atomic displacement parameters (Å^2^)

**Table:**

	*U*^11^	*U*^22^	*U*^33^	*U*^12^	*U*^13^	*U*^23^
O1	0.0205 (5)	0.0250 (6)	0.0405 (8)	−0.0007 (5)	−0.0004 (6)	−0.0028 (6)
O2	0.0553 (14)	0.0318 (11)	0.0324 (11)	−0.0147 (10)	0.000	0.000
N1	0.0206 (6)	0.0206 (7)	0.0234 (7)	−0.0014 (5)	0.0004 (6)	0.0008 (6)
C3	0.0240 (8)	0.0215 (8)	0.0246 (8)	0.0035 (6)	0.0014 (7)	0.0012 (8)
C2	0.0268 (8)	0.0193 (8)	0.0205 (7)	0.0009 (6)	−0.0014 (7)	0.0022 (7)
C4	0.0229 (8)	0.0208 (8)	0.0241 (9)	0.0003 (6)	−0.0011 (7)	0.0038 (8)
C7	0.0245 (8)	0.0243 (8)	0.0309 (10)	0.0025 (7)	−0.0008 (7)	−0.0041 (8)
C6	0.0244 (8)	0.0196 (8)	0.0213 (8)	0.0009 (6)	−0.0012 (7)	0.0019 (7)
C1	0.0309 (9)	0.0216 (8)	0.0284 (10)	−0.0005 (7)	−0.0007 (8)	−0.0012 (8)
C5	0.0255 (8)	0.0174 (7)	0.0258 (8)	−0.0004 (6)	−0.0006 (7)	0.0004 (7)

### Geometric parameters (Å, º)

**Table:**

O1—C4	1.283 (2)	C7—H7A	0.9800
O2—H2	0.862 (7)	C7—H7B	0.9800
N1—C2	1.362 (2)	C7—H7C	0.9800
N1—C6	1.359 (2)	C7—C6	1.498 (2)
N1—H1	0.871 (7)	C6—C5	1.367 (2)
C3—H3	0.9500	C1—H1A	0.9800
C3—C2	1.370 (2)	C1—H1B	0.9800
C3—C4	1.422 (3)	C1—H1C	0.9800
C2—C1	1.498 (2)	C5—H5	0.9500
C4—C5	1.428 (2)		
			
C2—N1—H1	116.8 (15)	C6—C7—H7A	109.5
C6—N1—C2	122.02 (14)	C6—C7—H7B	109.5
C6—N1—H1	120.9 (16)	C6—C7—H7C	109.5
C2—C3—H3	119.5	N1—C6—C7	116.70 (14)
C2—C3—C4	121.06 (16)	N1—C6—C5	119.79 (15)
C4—C3—H3	119.5	C5—C6—C7	123.49 (16)
N1—C2—C3	119.80 (16)	C2—C1—H1A	109.5
N1—C2—C1	116.63 (15)	C2—C1—H1B	109.5
C3—C2—C1	123.57 (16)	C2—C1—H1C	109.5
O1—C4—C3	122.52 (15)	H1A—C1—H1B	109.5
O1—C4—C5	121.34 (16)	H1A—C1—H1C	109.5
C3—C4—C5	116.14 (15)	H1B—C1—H1C	109.5
H7A—C7—H7B	109.5	C4—C5—H5	119.4
H7A—C7—H7C	109.5	C6—C5—C4	121.12 (16)
H7B—C7—H7C	109.5	C6—C5—H5	119.4
			
O1—C4—C5—C6	−179.53 (17)	C2—C3—C4—C5	−2.6 (3)
N1—C6—C5—C4	1.2 (3)	C4—C3—C2—N1	2.5 (3)
C3—C4—C5—C6	0.7 (3)	C4—C3—C2—C1	−176.66 (16)
C2—N1—C6—C7	177.28 (17)	C7—C6—C5—C4	−177.33 (17)
C2—N1—C6—C5	−1.4 (3)	C6—N1—C2—C3	−0.5 (3)
C2—C3—C4—O1	177.66 (16)	C6—N1—C2—C1	178.74 (17)

### Hydrogen-bond geometry (Å, º)

**Table:**

*D*—H···*A*	*D*—H	H···*A*	*D*···*A*	*D*—H···*A*
O2—H2···O1	0.86 (1)	1.96 (1)	2.8174 (17)	173 (2)
N1—H1···O1^i^	0.87 (1)	1.86 (1)	2.7154 (18)	166 (3)

Symmetry code: (i) *x*−1/2, −*y*+1/2, *z*.
