# Supplementary figures and images for: Crystal structure of 2,6-dimethyl-4-pyridone hemihydrate
Source: Acta Crystallogr E Crystallogr Commun. 2015 Jul 4;71(Pt 8):o533. doi: 10.1107/S2056989015012402 (PMC4571382; doi:10.1107/S2056989015012402)

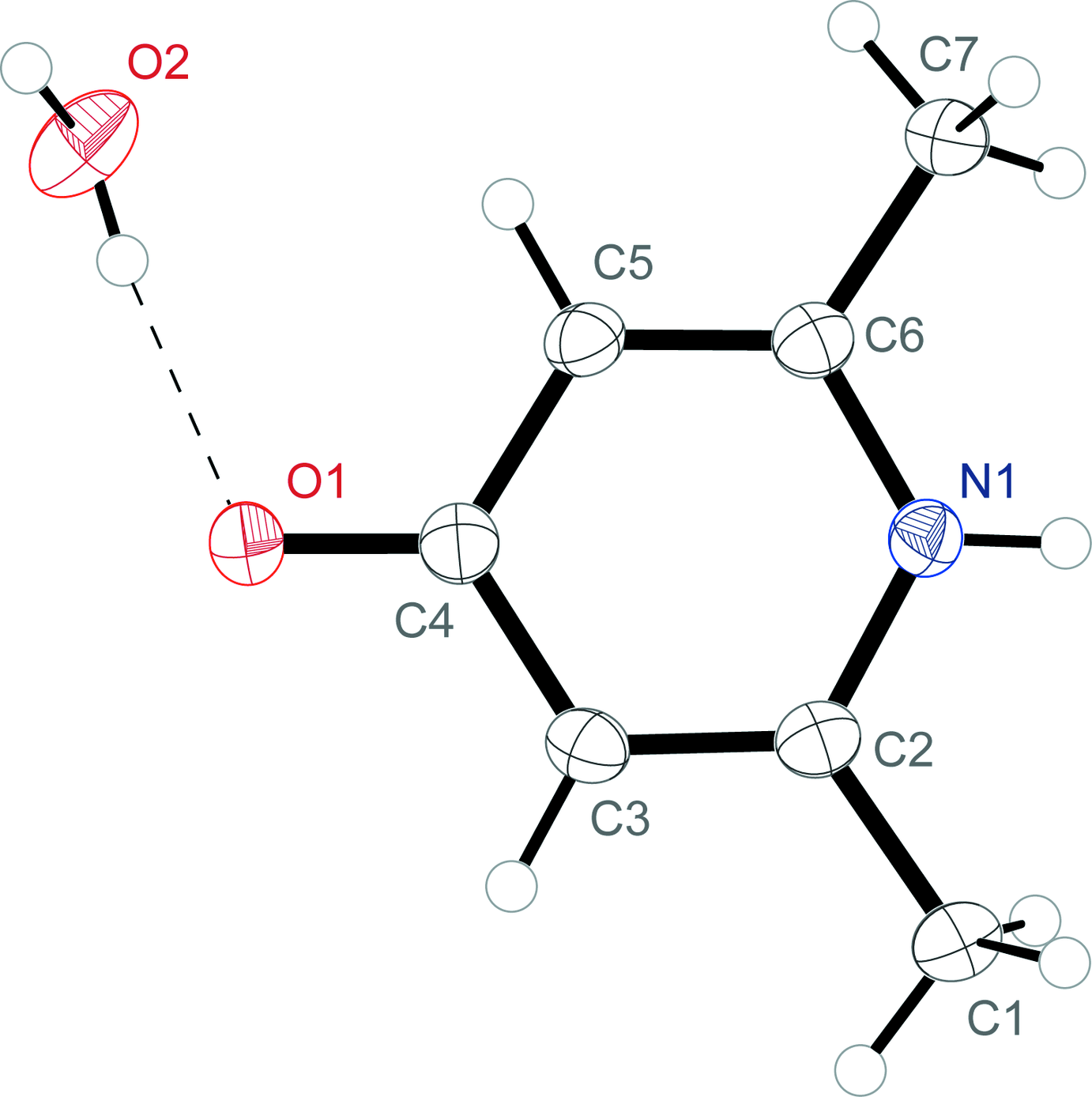

Supplement: Supplementary file 4 [file e-71-0o533-fig1.tif]

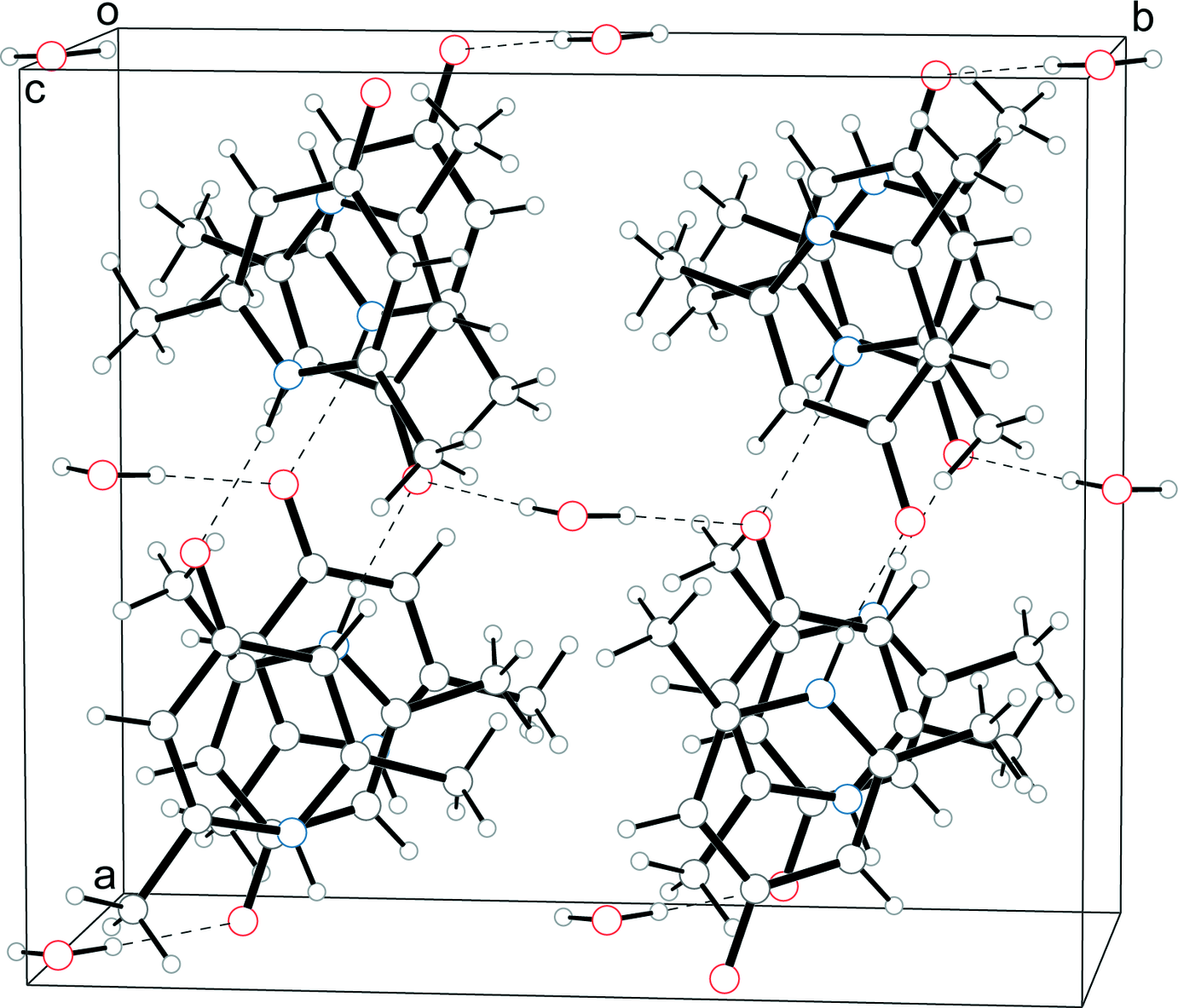

Supplement: Supplementary file 5 [file e-71-0o533-fig2.tif]
